# Chinese critical care certified course in intensive care unit: a nationwide-based analysis

**DOI:** 10.1186/s12909-023-04534-4

**Published:** 2023-08-15

**Authors:** Li Li, Qianghong Xu, Guolong Cai, Shijin Gong, Dawei Liu, Haibo Qiu, Kaijiang Yu, Dechang Chen, Xiangdong Guan, Jing Yan

**Affiliations:** 1 Department of Critical Care Medicine, Zhejiang Hospital, 12 Lingyin Road, Hangzhou, 310013 China; 2 Department of Critical Care Medicine, Peking Union Medical College Hospital, Peking Union Medical College, Chinese Academy of Medical Sciences, 1 Shuaifuyuan, Dongcheng District, Beijing, 100730 China; 3 Department of Critical Care Medicine, Zhongda Hospital, School of Medicine, Southeast University, Nanjing, 210009 China; 4 Department of Critical Care Medicine, The First Affiliated Hospital of Harbin Medical University, No. 23, Youzheng Road, Nangang District, Harbin, Heilongjiang China; 5 Department of Critical Care Medicine, Ruijin North Hospital, Shanghai Jiao Tong University School of Medicine, Shanghai, 201801 China; 6 Department of Critical Care Medicine, The First Affiliated Hospital of Sun Yat-sen University, Guangzhou, 510080 China

**Keywords:** Chinese Critical Care Certified Course, Intensive Care Unit Training, Training Program, Healthcare Improvement, Critical Care, Continuous Training

## Abstract

**Background:**

A training program for intensive care unit (ICU) physicians entitled “Chinese Critical Care Certified Course” (5 C) started in China in 2009, intending to improve the quality of intensive care provision. This study aimed to explore the associations between the 5 C certification of physicians and the quality of intensive care provision in China.

**Methods:**

This nationwide analysis collected data regarding 5 C-certified physicians between 2009 and 2019. Fifteen ICU quality control indicators (three structural, four procedural, and eight outcome-based) were collected from the Chinese National Report on the Services, Quality, and Safety in Medical Care System. Provinces were stratified into three groups based on the cumulative number of 5 C certified physicians per million population.

**Results:**

A total of 20,985 (80.41%) physicians from 3,425 public hospitals in 30 Chinese provinces were 5 C certified. The deep vein thrombosis (DVT) prophylaxis rate in the high 5 C physician-number provinces was significantly higher than in the intermediate 5 C physician-number provinces (67.6% vs. 55.1%, *p* = 0.043), while ventilator-associated pneumonia (VAP) rate in the low 5 C physician-number provinces was significantly higher than in the high 5 C physician-number provinces (14.9% vs. 8.9%, *p* = 0.031).

**Conclusions:**

The higher number of 5 C-certified physicians per million population seemed to be associated with higher DVT prophylaxis rates and lower VAP rates in China, suggesting that the 5 C program might have a beneficial impact on the quality of intensive care provision.

**Supplementary Information:**

The online version contains supplementary material available at 10.1186/s12909-023-04534-4.

## Background

Intensive care units (ICUs) provide specialized treatment for several serious life-threatening injuries and conditions, aiming to deliver continual day-to-day care with high-skill personnel and expert equipment. ICU physicians are required to make instantaneous decisions [1, 2] which may directly determine the patients’ survival. Despite constant improvements, medical errors or lower-quality of intensive care provision are still observed in 27-58% of ICU admissions [3] and are associated with higher ICU mortality [4].

Early diagnosis and intervention were shown to determine the prognosis of critical diseases such as sepsis, respiratory failure, or COVID-19 [5, 6]. Therefore, continuous training for ICU physicians in multiple disciplines is likely to improve their abilities to manage critical clinical conditions. The principle of formal up-to-date ongoing training is applied worldwide as one of the important ways to improve the quality of ICU medical care and reduce the mortality of the critically ill population [7–9]. Although some such training programs have been successfully utilized in sepsis or HIV/AIDS, there is still room for further improvement [10, 11].

In 2009 the Chinese Society of Critical Care Medicine and the Organization Management Department, and the Department of Continuing Education created a training model to standardize ICU care based on the American Society of Critical Care Medicine FCCS course (https://www.sccm.org/Fundamentals/Fundamental-Critical-Care-Support), the American Heart Association ECC course (https://cpr.heart.org/en), and the China Taiwan ACLS Joint Committee Courses (http://www.tsccm.org.tw/English/eng002.asp). The training program was named “Chinese Critical Care Certified Course (5 C)” (Supplementary File 1). For a decade (2009–2019), this program enrolled almost 20,000 clinicians in 31 provinces in China and provided multidisciplinary teams for training in an attempt to decrease ICU mortality [6, 12]. Our previous study reported that the increasing number of 5 C physicians was linked to lower COVID mortality [6], while another study reported controversial results for overall mortality [13]. The intended and actual effects of the 5 C certification program on other indicators of hospital care provision quality remain to be further discussed.

Based on the above, this study aimed to explore the associations between the 5 C certification of physicians and the quality of intensive care provision in China, focusing on the ICU quality indicators from Chinese national reports and analyzing its association with the number of 5 C physicians per million population.

## Methods

### Study design

This nationwide analysis enrolled physicians who participated in the Chinese 5 C training program (5 C physicians) between 2009 and 2019; ICU quality indicators were collected across China between 2015 and 2018. This study was approved by the Ethnic Committee of Zhejiang Hospital. Informed consent was waived by the Ethnic Committee due to the retrospective study design.

### 5 C training

The training consists of theoretical training and skill training. The theoretical training includes identification and assessment of severe patients, basic principles and methods of severe patient monitoring, hemodynamic monitoring and correction methods, shock, cardiopulmonary cerebral resuscitation, respiratory failure, chronic obstructive pulmonary disease and critical asthma, acute respiratory distress syndrome, mechanical ventilation, mechanical ventilation-related technologies, diagnosis and treatment of severe cardiovascular diseases, diagnosis and treatment of venous thrombosis and pulmonary embolism hemorrhage and coagulation disorders in severe patients, application principles of antibiotics in the ICU, fungal infection, catheter-related bloodstream infections, ventilator-associated pneumonia, sepsis and multiple organ dysfunction syndrome, acute liver injury and acute liver failure in severe patients, gastrointestinal failure in severe patients, enteral and parenteral nutrition support in severe patients, diagnosis and treatment of acute kidney injury, severe blood purification technology, analgesia and sedation of critically ill patients, critical diagnosis and treatment of central nervous system, internal environment disorder of critically ill patients, endocrine parameters and metabolism of critically ill patients, trauma, and scientific research in intensive care medicine. The skill training includes artificial airway establishment, mechanical ventilation, placement of vascular catheters, hemodynamic monitoring, and continuous renal replacement therapy techniques. The training is delivered face-to-face. The entire training duration is 4 days. The average size of an ICU is 20 beds, and they usually work as closed ICUs. Monthly classes can be held nationwide, in one place at a time. According to the official website of the Intensive Care Medicine Branch of the Chinese Medical Association, the website enrollment brochure, and online registration, the students choose their own time and location, at their convenience, to receive training and assessment.

### Data collection and definition

Data regarding the 5 C physicians were collected from the Chinese Society of Critical Care Medicine between 2009 and 2019, including their hospital, department, and province. A 5 C physician was defined as a clinician who had completed 5 C training and received 5 C certification. Since the ICU quality control survey in China was initiated in 2015, quality indicators between 2016 and 2018 were collected (including three structural, four procedural, and eight outcome-based) from the National Report on the Services, Quality, and Safety in Medical Care System issued by the National Health Commission of the People’s Republic of China. Specifically, the structural indicators were (1) the proportion of ICU patients to the total number of inpatients, (2) the proportion of ICU bed occupancy to the total inpatient bed occupancy, and (3) the proportion of patients with Acute Physiology, and Chronic Health Evaluation (APACHE) [14] II scores ≥ 15 to all ICU patients. The procedural indicators were (1) the 3-h Surviving Sepsis Campaign (SSC) bundle compliance rate [15], (2) the 6-h SSC bundle compliance rate [15], (3) the microbiology detection rate before antibiotics, and (4) deep vein thrombosis (DVT) prophylaxis rate. The outcome-based indicators were (1) unplanned endotracheal extubation rate, (2) reintubation rate within 48 h, (3) unplanned ICU transfer rate, (4) ICU readmission rate within 48 h, (5) ventilator-associated pneumonia (VAP) rate, (6) catheter-related bloodstream infection (CRBSI) rate, (7) catheter-associated urinary tract infection (CAUTI) rate, and (8) ICU mortality.

Population demographic data were derived from the 2020 China National Population Census, and provinces were stratified based on the cumulative number of 5 C physicians per million population and then divided into tertiles (three groups: low, intermediate, and high 5 C physician-number provinces). As the authors worked in Zhejiang province and data from many physicians were available in Zhejiang, data in Zhejiang province were sub-analyzed. Hospitals in Zhejiang were stratified according to the ratio of the number of physicians to ICU beds per hospital into 2 subgroups.

The gross domestic product (GDP) data in each province (except Taiwan) in China were collected from the China Statistical Yearbooks from the National Bureau of Statistics of the People’s Republic of China (available at: http://www.stats.gov.cn/tjsj/ndsj). Provinces were stratified according to GDP into 2 subgroups. The 5 C physician data and ICU quality indicators were compared in provinces with different GDPs and areas.

### Statistical analysis

Statistical analyses were performed utilizing SPSS 22.0 (IBM, Armonk, NY, USA) and GraphPad Prism 6 (GraphPad Software Inc., San Diego, CA, USA). Continuous variables with a normal distribution (according to the Kolmogorov-Smirnov test) were described as means ± standard deviations and were compared with the independent sample Student t-test between two groups or compared using one-way analysis of variance among three groups; continuous variables with skewed distributions were described as medians (quartiles) and were compared using Mann-Whitney U test between two groups or with Kruskal-Wallis H test and post hoc analysis among three groups. The categorical variables were described as n (%) and analyzed using the chi-square or Fisher’s exact test. The correlation between the cumulative number of 5 C certified physicians per million population and ICU quality indicators in each province was evaluated using the Spearman correlation analysis. All tests were two-sided, and *p*-values < 0.05 were considered statistically significant.

## Results

### 5 C certified physicians

**Fig. 1:**
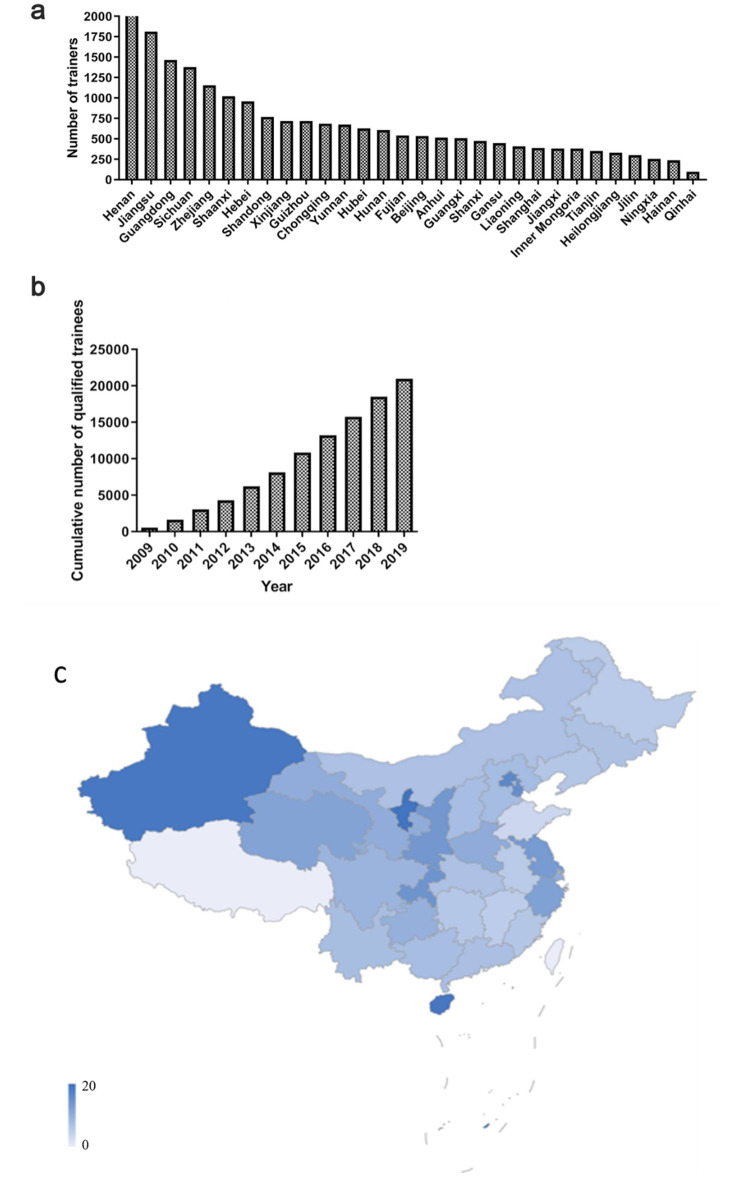
**Cumulative number of 5 C physicians per province in 2019 and number of 5 C certified physicians between 2009 and 2019 across China**. (**a**) Cumulative number of 5 C physicians in each province in 2019 across China. (**b**) Cumulative number of 5 C certified physicians between 2009 and 2019 across China. (**c**) Distribution of 5 C physicians per million population in China

One hundred twenty-eight training sessions were held in the 5 C training program between 2009 and 2019 with 26,099 physicians. The top three provinces with the highest number of 5 C physicians were Henan (9.59%), Jiangsu (8.63%), and Guangdong (6.99%), while Qinghai, Hainan, and Ningxia provinces had the fewest (Fig. 1a). As of 2019, a total of 20,985 physicians (80.41%) were 5 C certified, and the number of 5 C certified physicians trained each subsequent year has been steadily increasing, from 603 to 2009 to 3312 in 2019 (Fig. 1b); the passing rates varied from 72.7 to 90.5%. The top three provinces with the highest number of 5 C physicians per provincial population were Xinjiang, Gansu, and Hainan (Fig. 1c). A total of 13,158 (62.7%) of the 5 C-certified physicians worked in tertiary hospitals. In total, 15,319 5 C-certified physicians (73.0%) were working in the ICU department.

### Associations of 5 C certified physicians and ICU quality

A total of 3,425 ICUs reported quality indicators. There was no significant correlation between the cumulative number of 5 C certified physicians per million population and ICU quality indicators in each province (Data not shown). The DVT prophylaxis rate in the high 5 C physician-number provinces was significantly higher than in the intermediate 5 C physician-number provinces (67.6% vs. 55.1%, *p* = 0.043), and the VAP rate in the low 5 C physician-number provinces was significantly higher than in the high 5 C physician-number provinces (14.9% vs. 8.9%, *p* = 0.031) **(**Table 1**)**.

**Table 1:** ICU quality indicators in provinces with different number of 5 C physicians per million population

Indicators	Total	Low 5 C physician-number province (n = 10)	Intermediate 5 C physician-number province (n = 10)	High 5 C physician-number province (n = 10)	*p* values
Number of 5 C certified physicians	413 (270, 554)	311 (225,405)	446 (321,896)	481 (219,640)	0.107
Proportion of ICU in total inpatient bed occupancy (%)	2.0 (1.6,2.3)	1.7 (1.4,2.1)	2.1 (1.7,2.4)	2.0 (1.9,2.3)	0.342
Proportion of APACHE II score ≥ 15 in all ICU patients (%)	47.9 (41.7,53.7)	46.8 (41.7,50.8)	48.4 (39.9,51.5)	49.3 (47.1,53.7)	0.552
3-h SSC bundles compliance (%)	80.9 (71.1,85.7)	74.6 (73,82.5)	85.7 (71.1,88.8)	80.9 (69.8,85.7)	0.357
6-h SSC bundles compliance (%)	69.3 (57.6,75.4)	69.3 (60.1,73.9)	68.0 (56,78.4)	67.3 (57.6,77.4)	>0.999
Microbiology detection rate before antibiotics (%)	79.7 (78.5,83.8)	79.7 (73.6,79.7)	82.35 (79.7,83.8)	81.2 (79.7,85.8)	0.183
DVT prophylaxis rate (%)	56.9 (47.6,66.7)	54.5 (41.7,66.7)	55.1 (44.7,57.2)	67.6 (63.1,68.5)	0.043
Unplanned endotracheal extubation rate (%)	2.7 (1.6,3.5)	3.25 (2.1,6)	2.1 (1.4,3.1)	2.65 (1.6,3.5)	0.274
Reintubation rate within 48 h (%)	2.53 (1.97,3.76)	3.01 (2.51,3.85)	2.38 (1.88,4.03)	2.24 (1.88,3.49)	0.305
Rate of unplanned transfer to ICU (%)	9.6 (6.9,12.1)	8.9 (7.4,13.8)	9.8 (6.9,11.4)	8.9 (6.7,12.1)	0.736
ICU readmission rate within 48 h (%)	1.5 (1.2,2.3)	2.3 (1.3,2.6)	1.4 (1.1,1.7)	1.45 (1.1,1.7)	0.139
VAP rate (%)	10.8 (8.9,17.3)	14.9 (10.9,18.6)	10.5 (9.4,14.8)	8.9 (8.4,12.7)	0.031
CRBSI rate (%)	2.23 (1.71,2.23)	2.23 (2.23,4.46)	2.15 (1.37,2.23)	1.89 (1.37,2.23)	0.154
CAUTI rate (%)	3.39 (2.44,4.48)	3.60 (2.71,4.48)	3.39 (2.17,4.07)	3.19 (2.44,4.48)	0.792
ICU mortality (%)	8.8 (7.0,10.3)	9.5 (7.8,10.0)	8.8 (7.0,10.3)	8.4 (7.4,10.3)	0.646

Low 5 C physician-number province: the number of 5 C certified physicians per million population < 8.6; intermediate 5 C physician-number province: the number of 5 C certified physicians per million population ranged 8.6–13.0; High 5 C physician-number province: the number of 5 C certified physicians per million population ≥13.0

Abbreviations: ICU: intensive care unit; 5 C: Chinese Critical Care Certified Course; APACHE II: Acute Physiology and Chronic Health Evaluation II; SSC: Surviving Sepsis Campaign; CRBSI: catheter-related bloodstream infection; CAUTI: catheter-associated urinary tract infection; VAP: ventilator-associated pneumonia; DVT: deep vein thrombosis

### Changes in ICU quality

The cumulative number of 5 C certified physicians per million population has increased significantly from 7.8 (6.5, 12.7) in 2016 to 14.4 (9.9, 19.3) in 2018 (Table 2). The microbiology detection rate before antibiotics has also shown a significant increase from 70.52% (65.2%, 74.1%) in 2016 to 79.72% (78.29%, 83.8%) in 2018 (*p* < 0.001). The VAP rate has decreased from 15.39% (12.72%, 17.49%) in 2016 to 10.81% (8.90%, 17.42%) in 2018 (*p* = 0.014), and the CRBSI rate has also decreased [2.83% (2.23-3.6%) vs. 2.23% (1.63-2.66%), *p* = 0.022] (Table 2).

**Table 2:** ICU quality indicators in 2016 vs. 2018

Indicators	Year 2016, median (quartiles)	Year 2018, median (quartiles)	*p* values
Number of 5 C certified physician	335.00 (233.25, 430.75)	496.00 (350.50, 740.75)	0.009
5 C certified physician per million population	7.8 (6.5, 12.7)	14.4 (9.9, 19.3)	< 0.001
Proportion of APACEH II score ≥ 15 in all ICU patients (%)	52.38 (46.22, 58.00)	47.85 (41.69, 53.65)	0.071
3-h SSC bundles compliance (%)	76.77 (70.74, 82.80)	80.90 (71.06, 86.13)	0.589
6-h SSC bundles compliance (%)	65.71 (60.87, 72.97)	69.28 (57.18, 75.90)	0.847
Microbiology detection before antibiotics (%)	70.52 (65.20, 74.10)	79.72 (78.29, 83.80)	< 0.001
DVT prophylaxis rate (%)	62.53 (55.83, 67.59)	56.87 (46.90, 67.15)	0.187
Unplanned endotracheal extubation rate (%)	2.67 (1.69, 3.54)	2.67 (1.57, 3.60)	0.947
Reintubation rate within 48 h (%)	2.46 (2.04, 3.14)	2.53 (1.95, 3.78)	0.767
Rate of unplanned transfer to ICU (%)	8.06 (6.04, 11.25)	9.57 (6.88, 12.17)	0.314
ICU readmission rate within 48 h (%)	1.31 (0.99, 1.69)	1.53 (1.20, 2.26)	0.216
VAP rate (%)	15.39 (12.72, 17.49)	10.81 (8.90, 17.42)	0.014
CRBSI rate (%)	2.83 (2.23, 3.60)	2.23 (1.63, 2.66)	0.022
CAUTI rate (%)	3.80 (2.85, 5.43)	3.39 (2.44, 4.48)	0.083
ICU mortality (%)	8.94 (7.19, 10.69)	8.75 (6.81, 10.25)	0.378

Abbreviations: ICU: intensive care unit; 5 C: Chinese Critical Care Certified Course; APACHE II: Acute Physiology and Chronic Health Evaluation II; SSC: Surviving Sepsis Campaign; CRBSI: catheter-related bloodstream infection; CAUTI: catheter-associated urinary tract infection; VAP: ventilator-associated pneumonia; DVT: deep vein thrombosis

### Subgroup analysis of 5 C certified physicians and ICU quality in Zhejiang province

There were 1,151 5 C-certified physicians from 105 hospitals in Zhejiang Province in 2019. Hospitals were stratified as low 5 C physician number/ICU bed ratio subgroup (52 hospitals) and high 5 C physician number/ICU bed ratio subgroup (53 hospitals). The proportion of ICU in total inpatient bed occupancy in hospitals with low 5 C physician number/ICU bed ratio was significantly higher than those with high 5 C physician number/ICU bed ratio (2.47% vs. 2.00%, *p* < 0.001), while the DVT prophylaxis rate in hospitals with high 5 C physician number/ICU bed ratio was significantly higher than those with low 5 C physician number/ICU bed ratio (87.41% vs.79.16, *p* = 0.016) **(**Table 3**)**.

**Table 3:** ICU quality in Zhejiang subgroup in 2019

Indicators	Hospitals with low 5 C physician number / ICU bed ratio (n = 52)	Hospitals with high 5 C physician number/ICU bed ratio (n = 53)	*p* values
Number of 5 C certified physicians per hospital	8.00 (6.00, 10.55)	10.44 (7.000,16.82)	0.006
Number of ICU beds	20.00 (15.00,25.50)	17.00 (10.36,24.00)	0.097
Proportion of ICU in total inpatients (%)	1.58 (1.31,2.13)	1.44 (1.19,2.08)	0.199
Proportion of ICU in total inpatient bed occupancy (%)	2.47 (2.09,2.95)	2.00 (1.64,2.29)	< 0.001
Proportion of APACEH II score ≥ 15 in all ICU patients (%)	52.44 ± 20.53	51.85 ± 22.38	0.889
3-h SSC bundles compliance (%)	99.36 (89.67,100.00)	100.00 (90.08,100.00)	0.577
6-h SSC bundles compliance (%)	95.16 (76.22,100.00)	95.56 (76.39,100.00)	0.445
Microbiology detection before antibiotics (%)	98.67 (85.20,100.00)	99.75 (93.61,100.00)	0.237
DVT prophylaxis rate (%)	79.16 (47.76,94.38)	87.41 (66.49,98.57)	0.016
Unplanned endotracheal extubation rate (%)	0.63 (0.00,1.60)	0.48 (0.00,1.01)	0.452
Reintubation rate within 48 h (%)	1.83 (0.44,3.48)	1.67 (0.49,3.70)	0.772
Rate of unplanned transfer to ICU (%)	3.19 (0.24,15.55)	2.78 (0.00,17.72)	0.933
ICU readmission rate within 48 h (%)	0.71 (0.09,1.82)	0.71 (0.00,1.60)	0.497
VAP rate (%)	4.56 (3.32,8.24)	4.27 (1.99,6.66)	0.258
CRBSI rate (%)	1.39 (0.49,2.47)	1.37 (0.34,2.39)	0.693
CAUTI rate (%)	1.52 (0.57,3.13)	1.63 (0.68,3.83)	0.738
ICU mortality (%)	15.99 (11.30,23.03)	13.86 (10.67,19.18)	0.254

Abbreviations: ICU: intensive care unit; 5 C: Chinese Critical Care Certified Course; APACHE II: Acute Physiology and Chronic Health Evaluation II; SSC: Surviving Sepsis Campaign; CRBSI: catheter-related bloodstream infection; CAUTI: catheter-associated urinary tract infection; VAP: ventilator-associated pneumonia; DVT: deep vein thrombosis

### 5 C-certified physicians and ICU quality in different areas

There was no significant difference in the number of 5 C certified physicians per million population between the low-GDP provinces [10.8 (7.2, 12.5)] and high-GDP provinces [9.1 (8.2, 17.0)] (*p* = 0.694), and ICU quality indicators were similar between the 2 subgroups (Supplementary Table S1). The median number of 5 C certified physicians per million population in the east, northeast, southwest, northwest, and central areas of China per province were 1.3 (0.8–1.8), 0.7 (0.7–0.8), 1.5 (1.1–2.2), 0.9 (0.9–1.4) and 0.6 (0.6–0.8), respectively, with significant difference (*p* = 0.029) (Supplementary Table S2). The VAP rates in the east, northeast, southwest, northwest, and central areas of China per province were significantly different (9.15% vs. 15.00% vs. 15.26% vs. 8.90% vs. 14.75%, *p* = 0.041) (Supplementary Table S2).

## Discussion

Intensive care medicine in China has the same disciplinary status as internal medicine and surgery, which belong to secondary disciplines. After obtaining the physician qualification, physicians planning to work in the ICU should receive the 5 C training and a qualification certificate. The present study revealed a significant association between the higher numbers of 5 C certified physicians per million population and a higher DVT prophylaxis rate, as well as a lower VAP rate, highlighting the importance of the nationwide 5 C training program for improving the critical care provision quality.

In 2017, the World Health Organization recognized the value of training initiatives as a global health priority, highlighting its importance in improving care outcomes [16]. It has been reported that proper training, more reliable ultrasound, and clinical microbiology practices are essential for the early detection of sepsis [5, 17]. In this study, the 3-h SSC bundles compliance and 6-h SSC bundles compliance did not differ between the provinces with the different numbers of 5 C physicians per population (all *p* > 0.05). It could be partly explained by the comparable microbiology detection rate before antibiotics (*p* > 0.05) and other general characteristics. However, this analysis focused only on the qualification of the ICU physicians and did not consider the qualification and experience of the ICU nurses. Indeed, the experience of ICU nurses has been associated with the development of ICU sepsis [18]. In particular, Yousefi et al. [8] evaluated nurses’ knowledge before and after training, demonstrating improved quality of sepsis care. Nonetheless, the DVT prophylaxis rate was gradually increasing with the increase in the number of 5 C physicians per population, with a significant > 10% difference between the intermediate and high provinces, suggesting that better training of physicians is conducive to the individualization of clinical practice and might potentially lead to better sepsis and DVT outcomes.

Multidisciplinary knowledge is key to successful ICU pneumonia prevention [12]. Previous studies have confirmed that implementing robust multidisciplinary training improves overall intensive care provision quality [19, 20] and has been proven effective in optimizing the quality of anesthesia-associated care in particular [21, 22]. Of note, ICU teams led by intensivists or physicians trained in critical care have been shown to improve VAP outcomes [23, 24]. In line with the above research, this study demonstrated that the cumulative number of 5 C certified physicians per million population has increased almost two-fold between 2016 and 2018, while the VAP rate has decreased; with that VAP rate in the provinces with the low tertile number of 5 C physicians was almost 1.5 higher than in the high 5 C physician-number provinces. Prevention of VAP is the focus in ICU, especially for immunocompromised patients, older adults, and postoperative patients [25]. Obtained results suggest that 5 C training improves physicians’ knowledge and skills, leading to enhanced team performance that might prevent VAP with higher success.

Although adequate personnel training is vital for the successful management of the ICU, a previous study, finished in 2020 [13], failed to find an association between 5 C training and the reduction of ICU mortality. Currently, 5 C is the only national professional qualification training program in China that introduces standardized textbooks and standard courseware in clinical medicine, providing a continuing education model for other disciplines. The program is based on theoretical training taught in modules through theoretical key points, case discussions, questions and answers, and skill training. Some improvements are discussed based on the results of this study, including the revision of the 5 C textbook. The teaching PowerPoint has been continuously improved and revised to form an exceptional standardized course component. The form of theoretical training is more diversified and includes case discussions with questions, with answers added to the initial theoretical teaching. Conversely, physicians’ feedback can help improve the program and should be considered, while additional training methods should be explored for the next revision of the 5 C program, including the simulation-based training that recently demonstrated satisfactory results [26].

One of the goals for improving national medical quality and safety in 2022 and 2023 is to increase the standardized prevention rate of venous thromboembolism. The core strategy is as follows: (1) Medical institutions should establish a VTE prevention and control system within the hospital, a VTE management team composed of medical, clinical, nursing, and other departments, develop a scientific VTE prevention and control management path and carry out standardized VTE risk assessment and prevention. (2) Information technology should be used to strengthen VTE prevention reminders, data collection, monitoring, and evaluation feedback of quality control indicators and incorporate them into performance management, establishing incentive and constraint mechanisms. (3) Effective mass management tools should be used to find and analyze the factors that affect the organization to achieve this goal and to propose and implement continuous improvement measures. (4) VTE-related consultation, referral mechanisms, and emergency plans should be designed to achieve the treatment and management of severe VTE patients. (5) The hospital should implement VTE prevention and control technology guidance, teaching training, and related exchanges to improve VTE awareness and standardized prevention and control capabilities.

A major strength of the present study is its nationwide design and large sample size. This study covered the 11-year consecutive annual 5 C training data in 30 provinces (except Hong Kong, Macao, Xizang, and Taiwan) in China and data from the National Report of ICU quality control indicators. In addition, this is the first study to explore the impact of the cumulative number of 5 C-certified physicians on ICU quality to date, and the results are noteworthy as they highlighted the beneficial role of the nationwide 5 C training program on ICU quality improvement.

However, the present study is not without limitations. Firstly, the study was conducted based on generalized hospital data, not specific patient-based data. Secondly, the 5 C physicians were not evaluated for their individual knowledge and skills in critical care, and only the certification rate was available. Thirdly, the quality control indicators of the unit of each physician and the patients treated cannot correspond in a one-to-one way. In addition, some participating hospitals had very low numbers of physicians, which could lead to non-different outcomes in those hospitals. Fourthly, an interesting decline in CRBSI was observed between 2016 and 2018, but the reasons can be very complex (e.g., strengthened hospital infection prevention, improved maintenance of venous catheters, improved training, etc.), and the available data for the present study do not allow determining the exact reasons. Finally, the effect of the 5 C physicians may not be immediate and require long-term evaluation.

An important point to underline is that the present study demonstrates the difficulties inherent in evaluating a complex intervention like the 5 C educational program regarding its downstream effects on a large population of ICU patients. One of the strategies used here was to stratify the hospitals into tertiles of the proportion of 5 C-certified physicians to evaluate the intervention effect. Still, as shown in Table 1, even these proportions within tertiles are not significantly different from each other (P = 0.107). If, at the outset, the proportion of 5 C-certified physicians was homogenous between groups, it would be difficult to find correlations with better care.

## Conclusions

Provinces with a higher number of 5 C certified physicians per million population seem to be associated with higher DVT prophylaxis rates and lower VAP rates in China, suggesting that the 5 C program might have a beneficial impact on the quality of intensive care provision.

Abbreviation: 5 C: Chinese Critical Care Certified Course.

### Electronic supplementary material

Below is the link to the electronic supplementary material.

Supplementary Material 1

Supplementary Material 2

## List of Abbreviations

ICU: Intensive care unit
DVT: Deep vein thrombosis
APACHE: Acute Physiology, and Chronic Health Evaluation
SSC: Surviving Sepsis Campaign
VAP: Ventilator-associated pneumonia
CRBSI: Catheter-related bloodstream infection
CAUTI: Catheter-associated urinary tract infection
GDP: Gross domestic product
