# Supplementary material for: Chinese critical care certified course in intensive care unit: a nationwide-based analysis
Source: BMC Med Educ. 2023 Aug 15;23:576. doi: 10.1186/s12909-023-04534-4 (PMC10428552; doi:10.1186/s12909-023-04534-4)
Supplement: Supplementary file 1 — Supplementary Material 1 [file 12909_2023_4534_MOESM1_ESM.docx]

## Supplementary File 1. Training and assessment in Chinese Critical Care Certified course

The training content of Chinese Critical Care Certified course (5C) is divided into two parts: theory and skills. Theoretical training is based on the "Chinese Critical Care Medicine Specialty Qualification Training Textbook" (compiled by the Chinese Medical Association) and taught in modules. Each module is taught in the form of theoretical key points, case discussions, and questions and answers. The teaching content covers the identification and evaluation of critically ill patients, the basic principles and methods of critical illness monitoring, shock, cardiopulmonary cerebral resuscitation, the principles of application of antibacterial drugs in the ICU, enteral and parenteral nutrition support for critically ill patients, and analgesia and sedation. As of December 2019, the textbook had been revised three times. In addition to unified training materials, theoretical training also uses standard course materials. In order to ensure quality teaching, the teaching PowerPoint was developed with a unified format, standard courseware, and six revisions. Skills training include five modules: artificial airway establishment, mechanical ventilation, vascular catheter placement, hemodynamic monitoring, and continuous renal replacement therapy technology.

The training evaluation contains both theoretical and skill assessments. The theoretical assessment is performed on the last day of the 4-day theoretical training, and the skill assessment administered immediately thereafter. In order to ensure fairness, justice, and standardization of the assessment, it is organized and implemented by the Talent Exchange Service Center of the National Health Commission of the People’s Republic of China, from whom the assessment results are issued. Those who pass the assessment are issued a training certificate jointly signed by the Chinese Society of Critical Care Medicine, the Organization Management Department, and the Continuing Education Department.
