# Supplementary material for: Chinese critical care certified course in intensive care unit: a nationwide-based analysis
Source: BMC Med Educ. 2023 Aug 15;23:576. doi: 10.1186/s12909-023-04534-4 (PMC10428552; doi:10.1186/s12909-023-04534-4)
Supplement: Supplementary file 2 — Supplementary Material 2 [file 12909_2023_4534_MOESM2_ESM.docx]

**Supplementary Table S1. 5C physicians and ICU quality in provinces with different GDP in China**

| Indicators | Provinces with low GDP (n=15) | Provinces with high GDP (n=15) | *p* values |
| --- | --- | --- | --- |
| Number of 5C certified physicians per province | 422 (270, 554) | 405 (256,640) | 0.967 |
| Number of 5C certified physicians per million population | 10.8 (7.2, 12.5) | 9.1 (8.2, 17.0) | 0.694 |
| Proportion of ICU in total inpatient bed occupancy (%) | 1.95 (1.7, 2.4) | 2 (1.4,2.3) | 0.567 |
| Proportion of APACEH II score≥15 in all ICU patients (%) | 46.4 (34.4,58.4) | 48.6 (47.1,51.5) | 0.595 |
| 3-h SSC bundles compliance (%) | 79.3 (71.1,85.7) | 82.5 (69.8,87.6) | 0.935 |
| 6-h SSC bundles compliance (%) | 65.2 (60.1,78.4) | 71.3 (55,75.4) | 0.838 |
| Microbiology detection before antibiotics (%) | 80.9 (77.7,83.8) | 79.7 (78.5,83.8) | 0.436 |
| DVT prophylaxis rate (%) | 56.6 (47.6,65.5) | 64.3 (41.7,68.5) | 0.624 |
| Unplanned endotracheal extubation rate (%) | 3.1 (1.8,4.3) | 2.5 (1.6,3.1) | 0.217 |
| Reintubation rate within 48h (%) | 2.96 (1.97,4.03) | 2.42 (1.88,3.14) | 0.345 |
| Rate of unplanned transfer to ICU (%) | 9.7 (6.9,14.1) | 7.9 (6.9,12.1) | 0.806 |
| ICU readmission rate within 48 h (%) | 1.7 (1.2,2.3) | 1.3 (1.1,2.3) | 0.461 |
| VAP rate (%) | 12.7 (9.4,18.6) | 10.4 (8.9,14.8) | 0.325 |
| CRBSI rate (%) | 2.23 (1.71,2.23) | 2.23 (1.37,3.94) | 0.967 |
| CAUTI rate (%) | 3.53 (2.44,4.34) | 2.98 (2.44,4.48) | 0.653 |
| ICU Mortality (%) | 8.8 (6,10.3) | 8.5 (7,9.8) | 0.902 |

Abbreviations: ICU: intensive care unit; 5C: Chinese Critical Care Certified Course; APACHE II: Acute Physiology and Chronic Health Evaluation II; SSC: Surviving Sepsis Campaign; CRBSI: catheter-related bloodstream infection; CAUTI: catheter-associated urinary tract infection; VAP: ventilator-associated pneumonia; DVT: deep vein thrombosis.

**Supplementary Table S2. 5C physicians and ICU quality in different areas in China**

| Indicators | East area (n=10) | Northeast area (n=3) | Southwest area (n=6) | Northwest area (n=5) | Middle area (n=6) | *p* values |
| --- | --- | --- | --- | --- | --- | --- |
| Number of 5C certified physicians | 415.00 (291.25, 936.25) | 225.00 (208.00, 318.00) | 258.50 (143.75, 575.50) | 450.00 (431.50, 716.50) | 439.00 (308.25, 687.25) | 0.162 |
| Number of 5C physicians per million population | 13.4 (8.4, 18.5) | 7.4 (6.6, 8.2) | 15.3 (11.3, 22.6) | 10.8 (9.1, 14.3) | 7.9 (6.4, 10.0) | 0.029 |
| Proportion of ICU in total inpatient bed occupancy (%) | 2.25 (1.99, 2.44) | 1.50 (1.39, 1.95) | 1.80 (1.28, 2.09) | 2.07 (1.70,2.36) | 1.69 (1.54, 2.52) | 0.134 |
| Proportion of APACEH II score≥15 in all ICU patients (%) | 48.25±10.82 | 38.06±3.63 | 41.87±11.75 | 52.71±5.99 | 50.33±7.15 | 0.148 |
| 3-h SSC bundles compliance (%) | 75.32±12.05 | 78.25±9.16 | 74.55±6.98 | 86.04±4.86 | 80.90±5.21 | 0.104 |
| 6-h SSC bundles compliance (%) | 68.26 (55.78, 77.69) | 75.39 (61.13, 80.48) | 57.05 (51.45, 68.77) | 71.31 (57.30, 78.44) | 70.04 (66.98, 72.71) | 0.484 |
| Microbiology detection before antibiotics (%) | 79.71 (79.69, 83.80) | 67.45 (65.41, 79.72) | 80.53 (71.75, 86.05) | 83.80 (79.72, 93.80) | 79.92 (79.21, 84.83) | 0.274 |
| DVT prophylaxis rate (%) | 62.83 (52.85, 68.49) | 48.83 (25.01, 68.49) | 62.53 (47.20, 68.93) | 47.64 (43.77, 61.64) | 60.45 (49.88, 65.81) | 0.644 |
| Unplanned endotracheal extubation rate (%) | 1.77 (1.18, 3.30) | 6.29 (3.86, 11.48) | 2.87 (2.28, 3.44) | 2.75 (1.49, 3.34) | 2.16 (1.53, 4.15) | 0.089 |
| Reintubation rate within 48h (%) | 2.78±0.79 | 3.28±0.92 | 2.61±1.18 | 2.24±1.08 | 3.08±0.83 | 0.522 |
| Rate of unplanned transfer to ICU (%) | 8.98(6.84, 13.13) | 16.79(13.76, 23.50) | 8.82 (6.13, 11.20) | 6.71 (5.88, 9.90) | 8.90 (7.26, 10.91) | 0.061 |
| ICU readmission rate within 48 h (%) | 1.35 (1.08, 1.84) | 2.56 (1.29, 3.31) | 1.46 (1.23, 1.65) | 1.23 (0.84, 1.85) | 2.26 (1.44, 2.44) | 0.237 |
| VAP rate (%) | 9.15 (8.78,11.00) | 15.00 (14.75,19.33) | 15.26 (10.24, 19.20) | 8.90 (8.27,14.63) | 14.75 (10.05, 18.69) | 0.041 |
| CRBSI rate (%) | 2.23 (1.37, 2.23) | 4.11 (2.23, 6.51) | 2.06 (1.58, 4.74) | 1.71 (1.11, 2.06) | 2.23 (1.97, 4.54) | 0.109 |
| CAUTI (%) | 2.845 (2.54, 3.56) | 4.75 (2.98, 5.15) | 3.93 (3.15, 11.12) | 3.39 (1.83, 4.54) | 3.12 (2.03, 4.37) | 0.298 |
| ICU Mortality (%) | 8.14±2.07 | 11.00±2.10 | 8.58±2.24 | 9.20±2.40 | 7.90±3.31 | 0.424 |

Abbreviations: ICU: intensive care unit; 5C: Chinese Critical Care Certified Course; APACHE II: Acute Physiology and Chronic Health Evaluation II; SSC: Surviving Sepsis Campaign; CRBSI: catheter-related bloodstream infection; CAUTI: catheter-associated urinary tract infection; VAP: ventilator-associated pneumonia; DVT: deep vein thrombosis.
